# Complete Spectrum of Physical Comorbidities with Autism Spectrum Disorder in a Nationwide Cohort

**DOI:** 10.1007/s10803-024-06476-2

**Published:** 2024-07-27

**Authors:** Hans-Christoph Steinhausen, Martin Dalgaard Villumsen, René Klinkby Støving, Niels Bilenberg

**Affiliations:** 1 Department of Child and Adolescent Mental Health Odense, Mental Health Services in the Region of Southern Denmark, University of Southern Denmark, Odense, Denmark; 2 Department of Child and Adolescent Psychiatry, Psychiatric University Hospital of Zurich, Zurich, Switzerland; 3 Clinical Psychology and Epidemiology, Institute of Psychology, University of Basel, Basel, Switzerland; 4 Child and Adolescent Mental Health Centre, Capital Region Psychiatry, Copenhagen, Denmark; 5 Institute of Biological Psychiatry, Mental Health Center Sankt Hans, Roskilde, Denmark; 6 Center for Eating Disorders, Odense, University Hospital and Mental Health Services in the Region of Southern Denmark, Odense, Denmark

**Keywords:** Autism, Comorbidity, Physical disease, Nationwide study

## Abstract

The associations between autism spectrum disorder (ASD) and physical diseases (PD) based on ICD-8 and ICD-10 diagnoses were studied, comparing with the risks of the general population. All individuals diagnosed before 30th April 2018 with ASD (n = 12,063) and a 5% random sample of the general population (n = 41,251) were drawn from Danish registers of the birth cohorts 1984–1995. For each of the entire spectrum of 13 PD categories, participants were followed from birth to first diagnosis, death, emigration, or 31st December 2017, whichever came first. Time from inclusion at birth to time of first physical diagnosis, accounting for censoring, was studied by use of time-stratified Cox models. When compared to the control sample, the individuals with ASD had a substantial added immediate risk in infancy and in childhood for 12 of the 13 categories. Particularly prominent were estimated associations for nervous system diseases at ages 0–9, and diseases of the eye and adnexa at ages 0–11. The associations were observed for both sexes, but were stronger among females than males, especially for genitourinary system diseases. On the cumulative scale, individuals with ASD were at pronounced greater risk through follow-up for 8 categories, with the greatest cumulative risk of respiratory system diseases, which at ages 5 and 30 was 24.9% and 41.5% for the ASD cohort while for the control sample it was 16.3% and 34.5% at the same ages. Especially in infancy and childhood, the present study found increased risks for a multitude of physical diseases.

**Supplementary Information:**

The online version contains supplementary material available at 10.1007/s10803-024-06476-2.

## Introduction

According to recent reviews, international prevalence rates for autism spectrum disorder (ASD) indicate that some 2 per cent of children and young people are affected (Fombonne et al., 2021; Maenner et al., 2023). In a more restricted sample of 7- to 9-year olds in Denmark, the prevalence rate was estimated at 1.3 per cent (Delobel-Ayoub et al., 2020). With these prevalence rates, ASD is among the most frequent mental and developmental disorders. Given the dominance of genetic factors in the etiology of autism spectrum disorder (Bai et al., 2019) and the presence of a large number of pre- and perinatal risk factors (Gardener et al., 2009, 2011), conceptually, one has to consider that ASD exists from birth with varying time until the diagnosis is made. Furthermore, with half of the affected individuals experiencing a poor general outcome (Steinhausen et al., 2016) and varying impairments of adaptive functioning abilities (Chen et al., 2015; Levy et al., 2009), there is a strong need to study the entire life courses of the affected individuals in more detail.

In particular, the issue of the impact of comorbidity with both other mental disorders and physical diseases should be given more attention. So far, there is evidence of a higher prevalence of various physical diseases (PD) among patients with severe mental diseases than among the general population (De Hert et al., 2011; Momen et al., 2020). However, few studies have addressed the issue of the comorbidity between ASD and PD. A review of 10 studies indicated widespread co-occurring medical diseases among children and adolescents with ASD, but findings on some associations were inconsistent (Muskens et al., 2017). A recent umbrella review presented evidence for associations between ASD and rhinitis, obesity, and food allergy, only (Arrondo et al., 2022). In a case-comparison study, the most common medical diseases experienced by individuals with ASD were infections, obesity, neurological conditions, allergy and immunological conditions, musculoskeletal conditions and gastrointestinal conditions (Davignon et al., 2018). Building upon this study, a more recent paper reported that the most prevalent conditions in autistic youth at all ages were obesity, neurological disorders, anxiety, and ADHD (Malow et al., 2023). In hospital settings, the relative prevalence of several comorbidities among individuals with ASD were likewise much higher than for individuals without ASD. The overrepresented physical comorbidities included epilepsy, inflammatory bowel disease, bowel disorders, CNS/cranial abnormalities, diabetes mellitus type 1, and muscular dystrophy (Kohane et al., 2012). The association with epilepsy was also noted in a study based on Danish register data (Mouridsen et al., 2013).

So far, the risk of the entire range of PD for individuals with ASD has not been studied in representative populations across time and risk differences between the two sexes are not well described. The aim of this population-based study was to investigate the differences in immediate (i.e. on the hazard scale) and cumulative risk of PD for individuals with ASD and individuals of the Danish general population across time and between males and females.

### Methods

#### Participants

The study included 53,314 individuals from Danish population-based registers with identification carried out via the Danish Civil Registration System (CPR). Two samples were drawn from the 826,416 registered individuals born from 1984 to 1995: The 12,063 individuals who at follow-up on 30th April 2018 had been diagnosed with ASD as defined by the ICD-10 (World_Health_Organisation, 1992) and a 5% random sample of 41,251 individuals from the total population. ASD was defined as any of F84.0 (childhood autism), F84.1 (atypical autism), F84.5 (Asperger syndrome), or F84.8–9 (other ASD). The Lexis diagram in Online Resource 1 illustrates the numbers of first ASD-diagnosis for twelve 1-year birth cohorts from 1984 to 1995 by age. The diagram indicates an increasing number of individuals diagnosed during early adolescence (10–15 years) in the more recent birth cohorts. Individuals in both samples were followed for physical diagnoses from day of birth until the age of 33 years (oldest cohort) to 22 years (youngest cohort), unless censored because of emigration or death.

#### Procedure

Information on sex, date of birth, emigration, and death was available by the CPR while the national patient register (NPR) and the Danish psychiatric central research register (DPCRR) (Mors et al., 2011) provided data on physical diagnoses and on ASD diagnoses, respectively. Statistics Denmark, the central authority for Danish statistics, hosted and anonymized data for the study that ran under Danish Data Protection Agency (file 18/52623) approval. According to Danish legislation, ethical approval is not required for register-based studies.

#### Diagnoses

In Denmark, the ICD-8 (World_Health_Organisation, 1965) was used from 1969 to 1993, the ICD-9 was never adopted, and the ICD-10 (World_Health_Organisation, 1992) was used from 1994. In the interest of investigating cumulative incidences of comorbid PD, the analyses considered the complete list of PD following birth manifesting for the first time according to both the eighth and tenth ICD-classification. Analyses were performed using time to first disease after birth for each of the following major disease categories: (a) infectious diseases, (b) neoplasms, (c) blood diseases, (d) endocrine, nutritional, and metabolic diseases, (e) nervous system diseases, (f) diseases of the eye and adnexa, (g) diseases of the ear and mastoid process, (h) circulatory system diseases, (i) respiratory system diseases, (j) digestive system diseases, (k) skin and subcutaneous tissue diseases, (l) musculoskeletal system diseases, and (m) genitourinary system diseases. Online resource 2 represents the entire list of codes for PD as classified by both the ICD-10 and the ICD-8.

#### Statistical Analyses

For each of the 13 PD categories, using Cox proportional hazards models accounting for censoring, with time running from birth to first physical diagnosis, hazard ratios (HR) accompanied by 95% confidence intervals (CI) were estimated. The last day for physical follow-up was 31st December 2017. Separate effects were estimated on periods for which time-invariance was assessed with Aalen’s linear hazard models. Models were time stratified to obtain time invariance, which is a precondition for obtaining proportional hazards, giving varying HR estimates over time from inclusion, which was birth. For each outcome, which was a time-to-event, the stratification used gave estimates for age-periods specific to the outcome. The Cox models were adjusted for sex and the assumptions of proportional hazards were verified via Schoenfeld residuals tests. Tests for interaction between female and male (patients vs. reference) for PD were conducted. The method proposed by Holm was used to correct for multiple testing. The risk of each of the 13 disease categories over time for individuals with ASD and the references were compared using cumulative incidences, with death as competing risk, for the total and sex-stratified samples. Cumulative incidences were listed at ages 5, 10, 20, and 30. Analyses were performed with Stata 17.0 (Stata Corp, Texas, USA) on servers at Statistics Denmark.

## Results

**Fig. 1:**
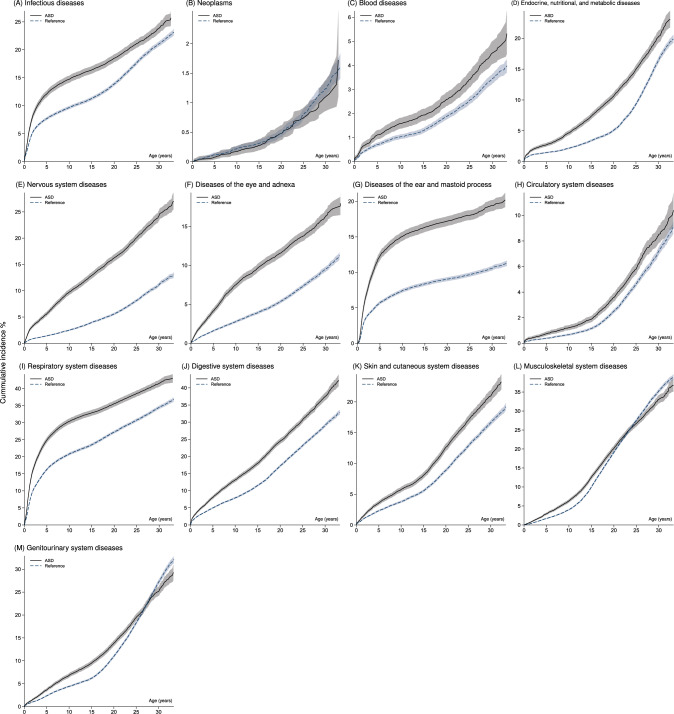
Cumulative incidence curves with pointwise confidence intervals (CI; shaded) of the corresponding PD category for the ASD and reference samples

There were 73.3% and 51.0% males among ASD-diagnosed and reference individuals, respectively, and death was observed for 117 (1.0%) and 521 (1.3%) individuals in the two samples. The Table in Online Resource 3 elaborates on status at physical follow-up for the 13 disease categories. Cumulative incidence curves (see Fig. 1) showed that individuals with ASD had higher cumulative risk across time for most disease categories, with the greatest risk increase for nervous system diseases and diseases of the ear and mastoid process. There were exceptions for neoplasms with no indication of difference across time, for musculoskeletal system diseases with a higher cumulative risk for the ASD-diagnosed individuals until an inversion at around age 22–24, and for genitourinary system diseases with a similar flip at age 26–28. The Table in Online Resource 4 shows differences in cumulative incidences at selected time points.

**Fig. 2:**
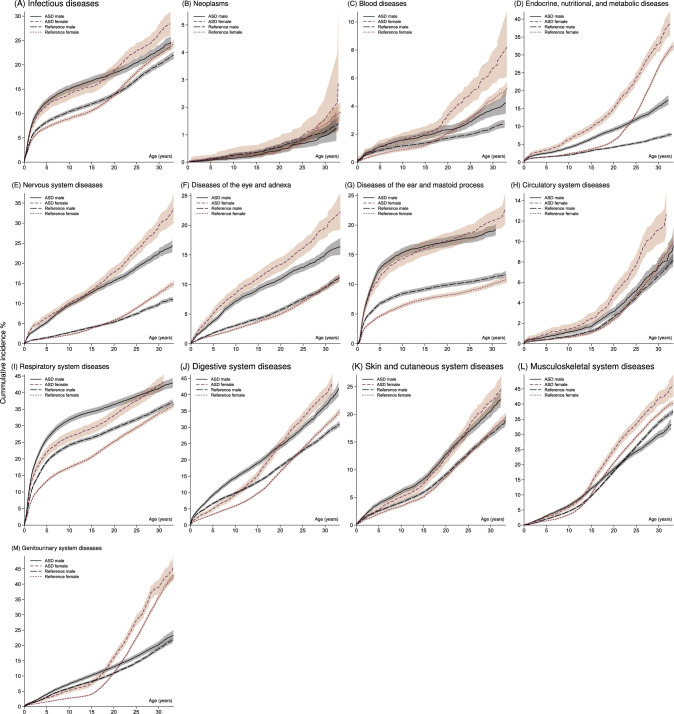
Sex-stratified cumulative incidence curves with pointwise confidence intervals (CI; shaded) of the corresponding PD category for the ASD and reference samples

When stratifying by sex, most cumulative incidence curves for both sexes (see Fig. 2) displayed higher risk through time for those diagnosed with ASD. Exceptions were for neoplasm with no sex-differences and for musculoskeletal system diseases, where the cumulative incidence after age 20–22 was higher for male references. The Table in Online Resource 5 collects sex-stratified cumulative incidences at selected time points.

Across sex, females were at higher cumulative risk after age 14–20 of infectious diseases, genitourinary system diseases, digestive system diseases, blood diseases, musculoskeletal system diseases, and nervous system diseases. For endocrine, nutritional, and metabolic diseases, females were at higher cumulative risk from birth onwards. For males, there was a greater risk of genitourinary system diseases and digestive system diseases during the first 16–18 years, while it was until age 22–24 for respiratory system diseases.

For all physical categories except neoplasms, the HR (see Table 1) for ASD-diagnosed compared to references, was 1.5 or greater for the first years in the total sample and in both sex-strata. For example, HR = 3.57 (95% CI, 3.21–3.97) and HR = 4.73 (95% CI, 4.10–5.47) for males and females, respectively, for nervous system diseases during the first nine years. The ratios had reduced after childhood. For most categories other than neoplasms, however, the immediate risk for individuals with ASD for each sex still was no smaller than for reference individuals. The two exceptions found were among males aged 16 years or older for infectious diseases with HR = 0.86 (95% CI, 0.78–0.94) and for musculoskeletal system diseases with HR = 0.73 (95% CI, 0.69–0.79).

**Table 1:** Hazard ratios (HR) (95% CI) of somatic diseases (first diagnosis) for individuals with ASD compared to reference individuals with mark for interactions by sex, and HR (95% CI) for females compared to males

	Total sample (patient vs. reference)	Males (patient vs. reference)	Females (patient vs. reference)	Patients (female vs. male)	Reference (females vs. males)	Interaction^a^
	HR (95% CI)	HR (95% CI)	HR (95% CI)	HR (95% CI)	HR (95% CI)	
Infectious diseases						
Age: 0–4	1.56 (1.46–1.66)	1.52 (1.41–1.64)	1.64 (1.47–1.84)	0.93 (0.82–1.04)	0.86 (0.80–0.92)	^b^
Age: 5–15	1.21 (1.09–1.33)	1.23 (1.09–1.38)	1.17 (0.98–1.40)	0.91 (0.75–1.10)	0.95 (0.87–1.05)	^b^
Age: 16 +	0.86 (0.78–0.94)	0.76 (0.68–0.86)	1.01 (0.88–1.15)	1.95 (1.66–2.29)	1.47 (1.37–1.58)	^b^
Neoplasms						
Age: 0–19	0.95 (0.70–1.28)	0.79 (0.55–1.14)	1.44 (0.87–2.37)	1.37 (0.79–2.37)	0.75 (0.56–1.00)	^b^
Age: 20 +	0.92 (0.66–1.27)	0.89 (0.58–1.36)	0.96 (0.58–1.59)	1.68 (0.92–3.06)	1.55 (1.19–2.03)	^b^
Blood diseases						
Age: 0–14	1.47 (1.25–1.72)	1.38 (1.14–1.67)	1.67 (1.26–2.20)	1.04 (0.78–1.39)	0.86 (0.73–1.03)	^b^
Age: 15 +	1.53 (1.31–1.80)	1.59 (1.25–2.03)	1.49 (1.21–1.84)	2.87 (2.19–3.75)	3.06 (2.58–3.63)	^b^
Endocrine, nutritional, and metabolic diseases						
Age: 0–6	2.08 (1.83–2.36)	1.86 (1.58–2.19)	2.48 (2.03–3.03)	1.39 (1.13–1.72)	1.04 (0.90–1.21)	^b^
Age: 7–17	2.63 (2.38–2.90)	2.45 (2.15–2.80)	2.87 (2.48–3.32)	1.65 (1.42–1.93)	1.41 (1.25–1.60)	^b^
Age: 18–24	1.74 (1.58–1.91)	2.55 (2.17–3.00)	1.42 (1.26–1.61)	3.21 (2.73–3.77)	5.76 (5.08–6.53)	p < 0.001
Age: 25 +	1.25 (1.10–1.44)	2.51 (2.00–3.16)	0.90 (0.75–1.08)	3.47 (2.71–4.45)	9.71 (8.33–11.3)	p < 0.001
Nervous system diseases						
Age: 0–9	3.94 (3.62–4.30)	3.57 (3.21–3.97)	4.73 (4.10–5.47)	1.05 (0.93–1.20)	0.79 (0.70–0.90)	^b^
Age: 10–13	2.40 (2.06–2.78)	2.50 (2.08–3.02)	2.20 (1.70–2.85)	0.95 (0.73–1.23)	1.08 (0.90–1.30)	^b^
Age: 14 +	2.10 (1.95–2.26)	2.02 (1.83–2.23)	2.20 (1.97–2.46)	1.69 (1.49–1.91)	1.55 (1.43–1.68)	^b^
Diseases of the eye and adnexa						
Age: 0–11	2.65 (2.44–2.88)	2.33 (2.10–2.58)	3.38 (2.94–3.87)	1.22 (1.07–1.39)	0.84 (0.75–0.93)	p < 0.001
Age: 12–20	1.53 (1.36–1.72)	1.41 (1.23–1.63)	1.79 (1.47–2.17)	1.23 (1.00–1.51)	0.97 (0.86–1.10)	^b^
Age: 21 +	1.22 (1.07–1.39)	1.05 (0.89–1.25)	1.56 (1.27–1.92)	1.67 (1.32–2.13)	1.13 (1.01–1.27)	^b^
Diseases of the ear and mastoid process						
Age: 0–4	2.06 (1.93–2.20)	1.91 (1.77–2.07)	2.50 (2.21–2.82)	0.88 (0.79–1.00)	0.68 (0.62–0.74)	^b^
Age: 5–14	1.72 (1.54–1.92)	1.66 (1.45–1.91)	1.82 (1.53–2.18)	1.26 (1.04–1.52)	1.15 (1.02–1.30)	^b^
Age: 15 +	1.57 (1.36–1.82)	1.31 (1.08–1.59)	2.04 (1.64–2.54)	1.88 (1.46–2.41)	1.20 (1.04–1.39)	^b^
Circulatory system diseases						
Age: 0–18	1.51 (1.33–1.70)	1.29 (1.11–1.51)	2.01 (1.65–2.45)	1.38 (1.12–1.70)	0.89 (0.78–1.01)	^b^
Age: 19 +	1.15 (1.03–1.29)	1.05 (0.91–1.21)	1.33 (1.12–1.59)	1.63 (1.33–1.99)	1.28 (1.15–1.41)	^b^
Respiratory system diseases						
Age: 0–3	1.52 (1.45–1.59)	1.44 (1.37–1.52)	1.76 (1.62–1.92)	0.83 (0.76–0.91)	0.68 (0.65–0.72)	^b^
Age: 4–9	1.27 (1.17–1.37)	1.25 (1.14–1.37)	1.32 (1.14–1.53)	0.75 (0.65–0.88)	0.71 (0.66–0.77)	^b^
Age: 10 +	1.00 (0.94–1.07)	0.98 (0.90–1.07)	1.03 (0.93–1.15)	1.49 (1.32–1.69)	1.42 (1.34–1.50)	^b^
Digestive system diseases						
Age: 0–4	1.42 (1.32–1.54)	1.40 (1.28–1.52)	1.53 (1.29–1.82)	0.54 (0.45–0.64)	0.49 (0.45–0.54)	^b^
Age: 5–10	1.71 (1.57–1.88)	1.72 (1.55–1.92)	1.69 (1.43–2.00)	0.80 (0.67–0.95)	0.81 (0.73–0.90)	^b^
Age: 11 +	1.33 (1.27–1.40)	1.28 (1.20–1.36)	1.42 (1.32–1.54)	1.49 (1.37–1.63)	1.34 (1.28–1.41)	^b^
Skin and cutaneous system diseases						
Age: 0–16	1.44 (1.34–1.54)	1.42 (1.31–1.55)	1.47 (1.30–1.67)	0.93 (0.82–1.06)	0.90 (0.84–0.97)	^b^
Age: 17 +	1.24 (1.15–1.33)	1.20 (1.10–1.31)	1.31 (1.16–1.48)	1.19 (1.04–1.36)	1.09 (1.02–1.17)	^b^
Musculoskeletal system diseases						
Age: 0–10	1.48 (1.36–1.61)	1.42 (1.29–1.56)	1.65 (1.42–1.91)	0.92 (0.79–1.07)	0.79 (0.72–0.86)	^b^
Age: 11–15	1.07 (0.99–1.16)	1.00 (0.90–1.11)	1.17 (1.04–1.32)	1.67 (1.45–1.92)	1.42 (1.32–1.53)	^b^
Age: 16 +	0.85 (0.80–0.89)	0.73 (0.69–0.79)	1.10 (1.01–1.20)	1.68 (1.52–1.85)	1.12 (1.08–1.18)	p < 0.001
Genitourinary system diseases						
Age: 0–14	1.36 (1.27–1.46)	1.24 (1.14–1.34)	1.89 (1.64–2.18)	0.73 (0.64–0.84)	0.48 (0.44–0.52)	p < 0.001
Age: 15–23	1.12 (1.05–1.21)	1.04 (0.94–1.17)	1.19 (1.08–1.30)	3.38 (2.98–3.82)	2.98 (2.77–3.19)	^b^
Age: 24 +	0.94 (0.84–1.05)	0.93 (0.79–1.09)	0.95 (0.82–1.10)	3.54 (2.89–4.32)	3.44 (3.15–3.75)	^b^

^a^Adjusted for multiple testing using Holm’s method

^b^p > 0.05

Table 1 also lists HRs for the comparison of females to males within each of the two samples. For both samples, females were at higher immediate risk among those getting the PD for the first time at about age 15 or older. The increased hazard among female individuals with ASD when compared to male individuals with ASD ranged from about 30% to about 250%, for example, HR = 2.87 (2.19–3.75) for blood diseases at age 15 or older. To the contrary, males were in both samples at about 30% and 40% higher immediate risk before the age of 10 with respect to respiratory system diseases. For reference individuals, females were at a 10% lower immediate risk before the age of 16 for skin and cutaneous system diseases (HR = 0.90 (0.84–0.97). For digestive system diseases, there was a higher hazard of about 50% at age 0–4 and of about 20% at age 5–10 for males in both samples. For genitourinary system diseases, the immediate risk for males was about 40% and 100% higher for ASD-diagnosed and references, respectively.

For females, compared to males, difference across samples in terms of an interaction pertained to the following conditions. Endocrine, nutritional, and metabolic diseases at age 18 or older with ratios above 1 that was greater among references than ASD-diagnosed. Diseases of the eye and adnexa at age 0–11 with an immediate risk among the ASD-diagnosed and the references that was greater and lower, respectively, for females. Musculoskeletal system diseases at age 16 or older with greater ratio above 1 among those with ASD than reference individuals and genitourinary system diseases at age 0–14 with greater immediate risk for females among the ASD-diagnosed than among the reference sample. Differences were most noticeable for endocrine, nutritional, and metabolic diseases; at the age of 18–24 HR = 3.21 (2.73–3.77) among individuals with ASD and HR = 5.76 (5.08–6.53) among reference individuals; at the age of 25 or older HR = 3.47 (2.71–4.45) and HR 9.71 (8.33–11.3), respectively.

## Discussion

In this study, for each of the 13 disease categories except neoplasms, the immediate risk of the first PD was at 50% or higher when comparing ASD-individuals to reference individuals during the early years of life. For both sexes the cumulative incidence of 8 out of the 13 disease groups were considerably greater among individuals with ASD than among the reference individuals from birth and onwards.

In both samples from some age and onwards, females were at greater cumulative risk than males. In contrast, males in both samples had a higher risk for respiratory and digestive system diseases, but only before the age of 10. In both samples, for those who reached age 10 + , 11 + , and 15 + , respectively without encountering the event, the hazard was greater for females than males for respiratory system diseases, digestive system diseases, musculoskeletal diseases, and genitourinary diseases, respectively.

Pre-existing studies are based on smaller data sets and lack coverage of the full spectrum of PD (Arrondo et al., 2022; Davignon et al., 2018; De Hert et al., 2011; Kohane et al., 2012; Mouridsen et al., 2013; Muskens et al., 2017). In contrast, the present study shows by extension that patients with ASD are subjected to a markedly increased immediate risk for the first diagnosis across the entire spectrum of PD except for neoplasms. Another recent Danish register study (Momen et al., 2020) found an increased physical vulnerability for most mental disorders including a broad category of developmental disorders without a specific focus on ASD.

In 8 out of 13 disease groups, irrespective of sex, the HR was greater than one at all time-points. The increased immediate risk even after childhood into adulthood showed that individuals with ASD who reached adulthood without getting the disease at hand were still at a considerably higher immediate risk of PD than the corresponding reference individuals. A thorough review of physical illness associated with the major mental disorders schizophrenia, bipolar illness, and major depression (Momen et al., 2020), argued that lifestyle and treatment specific factors account for much of the increased risk for most of these physical diseases. Future studies might also address the question whether or not there are common genetic factors accounting for both ASD and PD.

The overall higher immediate risk of females with ASD for most PD is remarkable in several ways, especially as this became evident in a developmental condition with a strong predominance of affected males amounting for almost three quarters of the present cohort. Interestingly, it matches findings from another recent register-based study by (blinded author name) in physical comorbidities subsequent to the diagnosis of anorexia nervosa. That study with a reversed sex distribution in terms of a greater female predominance found similar increased risks of PD (blinded reference). Accordingly, both studies found converging evidence for a generally heightened vulnerability of females for comorbid PD. Unfortunately, register-based studies with a limited range of data do not allow for further exploration of the extent to which this finding is rooted in the biology of sex or in gender-related behaviours in terms of a greater awareness of symptoms and a stronger tendency to seek professional medical assistance in females. Overall, the finding of an increased vulnerability for PD in females with ASD adds to the differences in the clinical phenomenology as evidenced by better cognitive development, less severe autistic symptoms, and a reduction of symptoms over time, but these difficulties in adaptive functioning and social challenges tend to emerge more for females in adolescence (Lai & Szatmari, 2020).

Among the strengths of the present study are the characteristics of the cohort design with large nationwide samples including individuals with ASD and a random sample of the general population included from birth to age 22 or longer. Free access to mental health care without individual costs as a part of the national health services and general practitioners referring patients with ASD mostly to psychiatry contribute to the representativeness of the samples. Furthermore, the findings of HRs were adjusted for sex. In addition, by design ASD were conditioned from birth allowing for time to a PD before first ASD-diagnosis. This procedure adds to the validity of the findings on the association of ASD diagnosed before age 22 up to 33 in young adulthood with PD.

Limitations include the structure of the NPR and DPCRR, which are registries not containing information at discharge or at the end of a treatment period needed for more adequate studies on the course of ASD. However, the focus of this study was on subsequent PD rather than on the outcome of ASD, which is rather stable according to clinical knowledge and outcome studies (Steinhausen et al., 2016). Furthermore, as ASD is classified differently in the ICD than in the DSM and the register-information on various subtypes of ASD is ambiguous, the association of subtypes of ASD with physical comorbidity was not tested.

In addition, the decision to define ASD only by use of the tenth ICD classification may have led to conservative findings. The eight ICD-revision did not contain any clear clinical descriptions and guidelines for diagnosis of ASD and served in Denmark until its replacement in 1994 by the tenth revision. Consequently, the diagnosis of ASD was inadequate in Denmark before 1994. Higher frequencies of initial ASD-diagnoses were found in younger ages with a peak around the age of 15 to 16 for the cohort born in 1995 and an only modest decline before the age of 22 at the end of follow up. The findings strongly suggest that the present ASD-sample is an underrepresentation of individuals who will receive a diagnosis before the age of 34 and possibly later. However, as the ASD-sample includes all individuals diagnosed with ASD before the age of 22, here is strong evidence that with complete identification of all individuals with ASD throughout the observation period, study results would be even more notable, resulting in greater HRs.

The fact that individuals with ASD in the study population were immune to any censoring (due to emigration or death), as illustrated by the Lexis diagram, before they were diagnosed with ASD, whereas the same did not apply to reference individuals, may have introduced a selection bias. However, only 3% of the references were lost due to emigration or death before age 22. For ASD-diagnosed, 1% were lost after diagnosis, but note that the immortal time bias for ASD individuals invalidates a comparison of death rates.

Concerns related to register-based studies are less limiting. There was no verification of the accuracy of diagnoses added to the DCPRR in this study. Prior quality checks of the DCPRR suggest high diagnostic validity for ASD (Lauritsen et al., 2010). As ASD was determined by treatment seeking and referral to hospital-based services, while not all with ASD may seek treatment, inference may not apply to untreated individuals with ASD. Furthermore, all patients were registered in the public health system. However, the rather small sector for psychiatrists in private practice is not required to register treated patients. Note finally, that information such as the severity, the degree of disability, and potential late effects of a disease are not available to national registers.

In terms of implications, this study provides the most comprehensive evidence so far that individuals with ASD, especially females, from birth to adulthood carry an increased risk for a large variety of physical diseases. The physical morbidity represents a major burden to the afflicted individuals and highlights the importance of an integrated medical supervision including the provision of the entire spectrum of general health care in addition to the focus of treatment of mental and developmental problems in individuals with autism. Furthermore, the results of the present study may serve as a background of future investigations into the still unknown etiological conditions of the associations of ASD with PD.

## Supplementary Information

Below is the link to the electronic supplementary material.

Online Resource 1: A Lexis diagram illustrating the number of first ASD-diagnoses according to ICD-10 for 12 Danish 1-year birth cohorts from 194 to 1995. Birth cohorts, which move along diagonal lines between calendar time on the horizontal axis and age on the vertical axis, were recorded in the DPCRR for ASD from 1994 onwards (between the vertical dashed lines). Individuals from the birth cohort 1984 reached an age from 33.3 to 34.3 (upper dashed line) at the end of follow-up while those from the 1995 birth cohort were younger than 23. The birth cohorts 1994 and 1995 were recordable for ASD from birth while older cohorts had a later entry with the age of 10 as an upper limit (lower dashed line). Supplementary file1 (PDF 14 KB)

Supplementary file2 (DOCX 14 KB)

Supplementary file3 (DOCX 17 KB)

Supplementary file4 (DOCX 18 KB)

Supplementary file5 (DOCX 21 KB)
