# Supplementary figures and images for: Complete Spectrum of Physical Comorbidities with Autism Spectrum Disorder in a Nationwide Cohort
Source: J Autism Dev Disord. 2024 Jul 27;55(11):3851–9. doi: 10.1007/s10803-024-06476-2 (PMC12575513; doi:10.1007/s10803-024-06476-2)

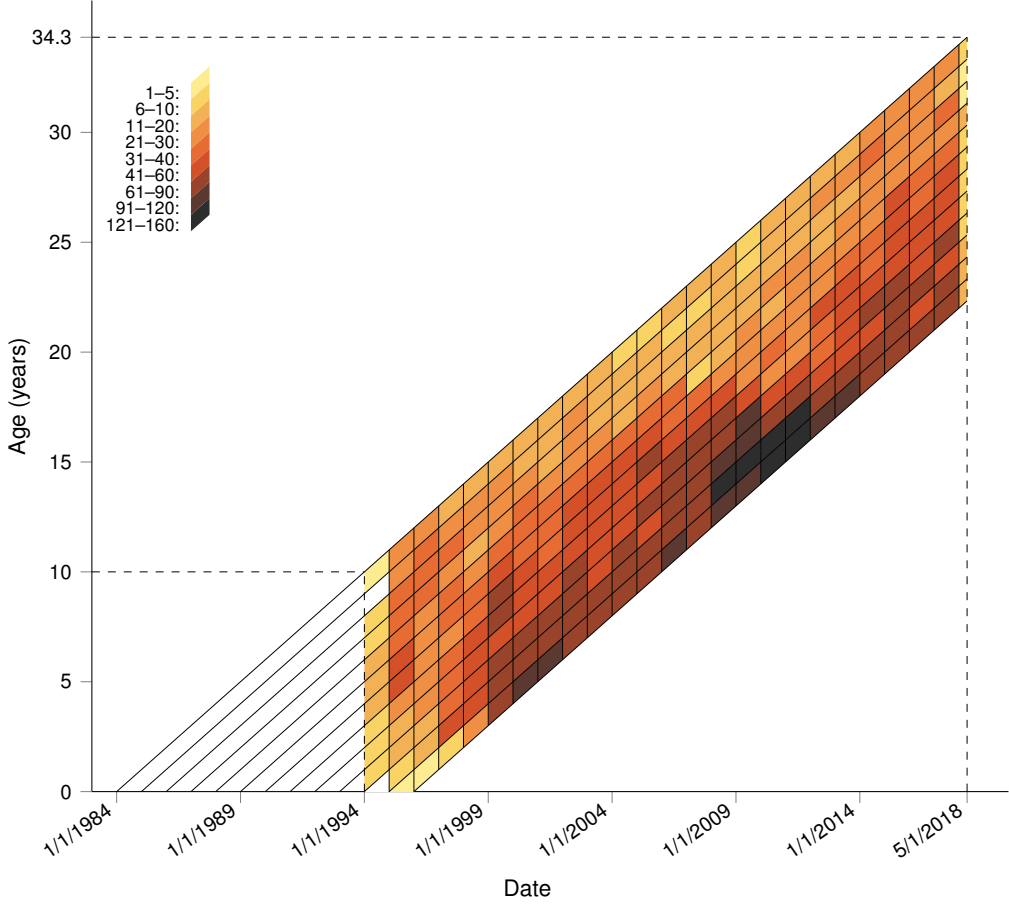

Supplement: Supplementary file 1 — Online Resource 1: A Lexis diagram illustrating the number of first ASD-diagnoses according to ICD-10 for 12 Danish 1-year birth cohorts from 194 to 1995. Birth cohorts, which move along diagonal lines between calendar time on the horizontal axis and age on the vertical axis, were recorded in the DPCRR for ASD from 1994 onwards (between the vertical dashed lines). Individuals from the birth cohort 1984 reached an age from 33.3 to 34.3 (upper dashed line) at the end of follow-up while those from the 1995 birth cohort were younger than 23. The birth cohorts 1994 and 1995 were recordable for ASD from birth while older cohorts had a later entry with the age of 10 as an upper limit (lower dashed line). Supplementary file1 (PDF 14 KB) [file 10803_2024_6476_MOESM1_ESM.pdf]
