# Supplementary material for: Complete Spectrum of Physical Comorbidities with Autism Spectrum Disorder in a Nationwide Cohort
Source: J Autism Dev Disord. 2024 Jul 27;55(11):3851–9. doi: 10.1007/s10803-024-06476-2 (PMC12575513; doi:10.1007/s10803-024-06476-2)
Supplement: Supplementary file 2 — Supplementary file2 (DOCX 14 KB) [file 10803_2024_6476_MOESM2_ESM.docx]

**Online resource 2.** Classification codes of physical disease categories in ICD-10 and ICD-8

| **Disease category** | **ICD-10** | **ICD-8** |
| --- | --- | --- |
| Infectious diseases | A00-A09; A15-A28; A30-A89; A92-A99; B00-B09; B15-B83; B85-B99 | 000-009; 010-019; 020-027; 030-039; 040-046; 050-057; 060-068; 070-079; 080-089; 090-099; 110-117; 120-129; 130-136 |
| Malignant neoplasms | C00-C97 | 140-149; 150-159; 160-163; 170-174; 180-189; 190-199 |
| Blood diseases | D50-D53; D55-D77; D80-D89 | 280-289 |
| Endocrine, nutritional and metabolic diseases | E00-E07; E10-E16; E20-E35; E40-E46; E50-E68; E70-E90 | 240-246; 250-258; 260-269; 270-279 |
| Nervous system diseases | G00-G14; G20-G26; G30-G32; G35-G37, G40-G47; G50-G64; G70-G73; G80-G83; G90-G99 | 320-324; 330-333; 340-349; 350-358 |
| Diseases of the eye and adnexa | H00-H59 | 360-369; 370-379; |
| Diseases of the ear and mastoid process | H60-H95 | 380-389 |
| Circulatory system diseases | I00-I02, I05-I15; I20-I28; I30-I52; I60-I99 | 390-392; 393-398; 400-404; 410-414; 420-429; 430-438; 440-448; 450-458 |
| Respiratory system diseases | J00-J06; J09-J18; J20-J22; J30-J47; J60-J70; J80-J99 | 460-466; 470-474; 480-486; 490-493; 500-508; 510-519 |
| Digestive system diseases | K00-K14; K20-K31; K35-K38; K40-K46; K50-K52; K55-K67; K70-K77; K80-K87; K90-K93 | 520-529; 530-537; 540-543; 544-544; 550-553; 560-569; 570-577 |
| Skin and subcutaneous tissue diseases | L00-L08; L10-L14; L20-L30; L40-L45; L50-L75; L80-L99 | 680-686; 690-698; 700-709 |
| Musculoskeletal system diseases | M00-M25; M30-M36; M40-M54; M60-M99 | 710-718; 720-729; 730-738 |
| Genitourinary system diseases | N00-N08; N10-N23; N25-N51; N40-N51; N60-N64; N70-N77; N80-N99 | 580-584; 590-599; 600-607; 610-616; 620-629 |
