# Supplementary material for: Complete Spectrum of Physical Comorbidities with Autism Spectrum Disorder in a Nationwide Cohort
Source: J Autism Dev Disord. 2024 Jul 27;55(11):3851–9. doi: 10.1007/s10803-024-06476-2 (PMC12575513; doi:10.1007/s10803-024-06476-2)
Supplement: Supplementary file 3 — Supplementary file3 (DOCX 17 KB) [file 10803_2024_6476_MOESM3_ESM.docx]

|  | **ASD** | | | | **Reference** | | | |
| --- | --- | --- | --- | --- | --- | --- | --- | --- |
|  | **Censored** | **Event** | **Death** | **Exclude** | **Censored** | **Event** | **Death** | **Exclude** |
| **Infectious diseases** | 9257 | 2598 | 68 | 140 | 32762 | 7828 | 348 | 313 |
| **Neoplasms** | 11854 | 102 | 107 | 0 | 40354 | 421 | 414 | 62 |
| **Blood diseases** | 11487 | 445 | 97 | 34 | 39479 | 1217 | 425 | 130 |
| **Endocrine, nutritional, and metabolic diseases** | 9894 | 1951 | 69 | 149 | 35299 | 5185 | 401 | 366 |
| **Nervous system diseases** | 9446 | 2535 | 63 | 19 | 36886 | 3914 | 355 | 96 |
| **Diseases of the eye and adnexa** | 10181 | 1735 | 81 | 66 | 37160 | 3435 | 420 | 236 |
| **Diseases of the ear and mastoid process** | 9757 | 2225 | 79 | 2 | 36635 | 4143 | 410 | 63 |
| **Circulatory system diseases** | 11171 | 793 | 77 | 22 | 38385 | 2399 | 377 | 90 |
| **Respiratory system diseases** | 7244 | 4726 | 47 | 46 | 27494 | 13258 | 315 | 184 |
| **Digestive system diseases** | 8065 | 3912 | 60 | 26 | 30189 | 10570 | 381 | 111 |
| **Skin and cutaneous system diseases** | 9800 | 2171 | 85 | 7 | 34759 | 5970 | 422 | 100 |
| **Musculoskeletal system diseases** | 8572 | 3419 | 63 | 9 | 28142 | 12645 | 392 | 72 |
| **Genitourinary system diseases** | 9448 | 2527 | 77 | 11 | 31557 | 9199 | 406 | 89 |

**Online Resource 3.** Status at somatic follow-up on 31^st^ December 2017 for the ASD and reference samples. For each of the 13 somatic disease categories, the event of interest is the first diagnosis after birth. Individuals were censored at follow-up or at emigration if the event had not been observed, or flagged for the event or for death, whichever occurred first. Individuals experiencing an event at the date of birth (time of day is not recorded in the NPR) were excluded from the corresponding risk set, which consisted of all subjects who were at risk of experiencing the event after birth. In total, death and emigration were observed for 117 (1.0%) and 171 (1.4%) of the ASD-diagnosed, respectively, while the frequencies for references were 521 (1.3%) and 2352 (5.7%).
