# Supplementary material for: Complete Spectrum of Physical Comorbidities with Autism Spectrum Disorder in a Nationwide Cohort
Source: J Autism Dev Disord. 2024 Jul 27;55(11):3851–9. doi: 10.1007/s10803-024-06476-2 (PMC12575513; doi:10.1007/s10803-024-06476-2)
Supplement: Supplementary file 4 — Supplementary file4 (DOCX 18 KB) [file 10803_2024_6476_MOESM4_ESM.docx]

|  | **Cumulative incidence % (95% CI)** | | | | **Cumulative incidence % (95% CI)** | | | |
| --- | --- | --- | --- | --- | --- | --- | --- | --- |
|  | **ASD group** | | | | **Reference group** | | | |
|  | At age 5 | At age 10 | At age 20 | At age 30 | At age 5 | At age 10 | At age 20 | At age 30 |
| **Infectious diseases** | 12.1 (11.5–12.7) | 14.7 (14.1–15.4) | 18.5 (17.8–19.2) | 23.8 (22.9–24.7) | 7.70 (7.45–7.96) | 9.67 (9.38–9.96) | 13.9 (13.5–14.2) | 21.0 (20.6–21.5) |
| **Neoplasms** | 0.0750 (0.0380–0.139) | 0.166 (0.105–0.253) | 0.473 (0.363–0.61) | 1.08 (0.848–1.36) | 0.115 (0.086–0.152) | 0 .220 (0.179–0.27) | 0.477 (0.413–0.548) | 1.23 (1.11–1.37) |
| **Blood diseases** | 1.11 (0.931–1.31) | 1.58 (1.37–1.81) | 2.59 (2.32–2.88) | 4.51 (4.05–5.00) | 0.705 (0.628–0.790) | 1.03 (0.936–1.13) | 1.88 (1.75–2.01) | 3.45 (3.25–3.66) |
| **Endocrine, nutritional, and metabolic diseases** | 2.74 (2.46–3.05) | 4.65 (4.28–5.04) | 10.6 (10.1–11.2) | 20.2 (19.2–21.2) | 1.47 (1.36–1.59) | 2.17 (2.03–2.32) | 5.10 (4.88–5.31) | 16.4 (16.0–16.9) |
| **Nervous system diseases** | 5.57 (5.17–5.99) | 9.71 (9.19–10.2) | 16.2 (15.6–16.9) | 24.2 (23.3–25.2) | 1.4 (1.29–1.52) | 2.49 (2.34–2.64) | 5.59 (5.37–5.81) | 11.1 (10.8–11.5) |
| **Diseases of the eye and adnexa** | 4.13 (3.79–4.50) | 7.51 (7.05–7.99) | 11.6 (11.0–12.2) | 16.2 (15.4–17.0) | 1.6 (1.49–1.73) | 2.78 (2.62–2.94) | 5.39 (5.17–5.61) | 9.52 (9.19–9.85) |
| **Diseases of the ear and mastoid process** | 12.3 (11.7–12.9) | 15.0 (14.4–15.7) | 17.2 (16.5–17.9) | 19.4 (18.6–20.1) | 5.73 (5.51–5.96) | 7.41 (7.16–7.67) | 9.03 (8.75–9.31) | 10.6 (10.3–10.9) |
| **Circulatory system diseases** | 0.747 (0.606–0.914) | 1.21 (1.03–1.42) | 3.58 (3.26–3.92) | 8.41 (7.76–9.08) | 0.392 (0.336–0.457) | 0.668 (0.593–0.751) | 2.50 (2.35–2.65) | 7.16 (6.86–7.47) |
| **Respiratory system diseases** | 24.9 (24.1–25.6) | 30.4 (29.6–31.2) | 35.6 (34.7–36.4) | 41.5 (40.5–42.5) | 16.3 (15.9–16.6) | 20.8 (20.4–21.2) | 27.3 (26.8–27.7) | 34.5 (34.0–35.0) |
| **Digestive system diseases** | 8.05 (7.57–8.55) | 13.1 (12.5–13.7) | 24.5 (23.7–25.2) | 37.5 (36.4–38.6) | 4.98 (4.77–5.19) | 7.91 (7.65–8.17) | 17.2 (16.9–17.6) | 29.1 (28.6–29.6) |
| **Skin and cutaneous system diseases** | 3.57 (3.25–3.92) | 5.77 (5.37–6.20) | 12.6 (12.0–13.2) | 20.9 (20.0–21.9) | 2.31 (2.17–2.46) | 3.78 (3.60–3.97) | 8.88 (8.61–9.16) | 16.6 (16.2–17.0) |
| **Musculoskeletal system diseases** | 2.92 (2.63–3.23) | 6.33 (5.91–6.78) | 20.1 (19.4–20.9) | 33.0 (32.0–34.1) | 1.69 (1.57–1.82) | 4.03 (3.84–4.22) | 19.1 (18.7–19.5) | 35.3 (34.7–35.8) |
| **Genitourinary system diseases** | 3.63 (3.31–3.98) | 6.80 (6.36–7.26) | 13.8 (13.2–14.5) | 25.2 (24.2–26.2) | 2.34 (2.20–2.49) | 4.41 (4.22–4.61) | 11.0 (10.7–11.3) | 27.2 (26.7–27.7) |

**Online Resource 4.** Cumulative incidences of somatic diseases for the two study groups
