# Supplementary material for: Complete Spectrum of Physical Comorbidities with Autism Spectrum Disorder in a Nationwide Cohort
Source: J Autism Dev Disord. 2024 Jul 27;55(11):3851–9. doi: 10.1007/s10803-024-06476-2 (PMC12575513; doi:10.1007/s10803-024-06476-2)
Supplement: Supplementary file 5 — Supplementary file5 (DOCX 21 KB) [file 10803_2024_6476_MOESM5_ESM.docx]

## **Online Resource 5.** Sex stratified cumulative incidences of somatic diseases in the two study groups

|  | Cumulative incidence % (95% CI) | | | | Cumulative incidence % (95% CI) | | | |
| --- | --- | --- | --- | --- | --- | --- | --- | --- |
| **Males** | **ASD group** | | | | **Reference group** | | | |
|  | At age 5 | At age 10 | At age 20 | At age 30 | At age 5 | At age 10 | At age 20 | At age 30 |
| **Infectious diseases** | 12.3 (11.7–13.0) | 15.0 (14.3–15.8) | 18.4 (17.6–19.2) | 22.9 (21.9–23.9) | 8.24 (7.87–8.62) | 10.4 (9.98–10.8) | 13.9 (13.4–14.4) | 20.1 (19.5–20.7) |
| **Neoplasms** | 0.068 (0.029–0.145) | 0.136 (0.075–0.234) | 0.431 (0.311–.587) | 0.918 (0.685–1.21) | 0.124 (0.084–0.181) | 0.255 (0.193–0.331) | 0.543 (0.45–0.651) | 1.09 (0.94–1.26) |
| **Blood diseases** | 1.10 (0.90–1.34) | 1.57 (1.32–1.84) | 2.31 (2.01–2.64) | 3.72 (3.25–4.23) | 0.827 (0.712–0.958) | 1.15 (1.01–1.30) | 1.70 (1.53–1.88) | 2.46 (2.23–2.71) |
| **Endocrine, nutritional, and metabolic diseases** | 2.50 (2.19–2.84) | 4.06 (3.66–4.48) | 9.02 (8.43–9.63) | 15.5 (14.5–16.5) | 1.48 (1.32–1.65) | 2.00 (1.81–2.19) | 4.21 (3.94–4.49) | 6.87 (6.48–7.27) |
| **Nervous system diseases** | 5.25 (4.80–5.73) | 9.6 (8.99–10.2) | 15.7 (14.9–16.4) | 22.3 (21.3–23.4) | 1.54 (1.38–1.71) | 2.77 (2.55–3.00) | 5.38 (5.08–5.70) | 9.73 (9.27–10.2) |
| **Diseases of the eye and adnexa** | 3.78 (3.40–4.19) | 7.2 (6.67–7.75) | 10.9 (10.3–11.6) | 15 (14.1–15.9) | 1.8 (1.63–1.99) | 3.07 (2.84–3.31) | 5.72 (5.40–6.04) | 9.55 (9.09–10.0) |
| **Diseases of the ear and mastoid process** | 12.7 (12.0–13.4) | 15.3 (14.6–16.1) | 17.2 (16.4–18.0) | 18.9 (18.0–19.8) | 6.77 (6.44–7.12) | 8.41 (8.04–8.79) | 9.87 (9.47–10.3) | 11.1 (10.7–11.6) |
| **Circulatory system diseases** | 0.681 (0.525–0.87) | 1.13 (0.93–1.37) | 3.14 (2.79–3.52) | 7.46 (6.75–8.22) | 0.396 (0.319–0.489) | 0.725 (0.617–0.847) | 2.64 (2.42–2.86) | 6.60 (6.20–7.01) |
| **Respiratory system diseases** | 26.0 (25.0–26.9) | 31.8 (30.8–32.8) | 36.4 (35.4–37.5) | 41.3 (40.1–42.4) | 19.1 (18.6–19.7) | 24.2 (23.6–24.7) | 29.2 (28.6–29.8) | 35.1 (34.4–35.8) |
| **Digestive system diseases** | 9.16 (8.57–9.77) | 14.5 (13.8–15.2) | 24.3 (23.4–25.2) | 36.6 (35.3–37.9) | 6.60 (6.27–6.94) | 9.84 (9.44–10.2) | 18.0 (17.5–18.5) | 27.8 (27.1–28.5) |
| **Skin and cutaneous system diseases** | 3.85 (3.46–4.27) | 6.03 (5.55–6.54) | 12.8 (12.1–13.5) | 20.5 (19.4–21.5) | 2.59 (2.38–2.81) | 4.12 (3.85–4.39) | 9.12 (8.73–9.51) | 16.4 (15.8–17.0) |
| **Musculoskeletal system diseases** | 3.02 (2.68–3.40) | 6.51 (6.00–7.03) | 18.5 (17.7–19.3) | 29.9 (28.6–31.1) | 1.97 (1.78–2.16) | 4.56 (4.29–4.85) | 17.7 (17.2–18.2) | 33.9 (33.1–34.6) |
| **Genitourinary system diseases** | 3.94 (3.55–4.36) | 7.37 (6.84–7.93) | 13.0 (12.3–13.7) | 20.2 (19.2–21.3) | 3.10 (2.87–3.34) | 6.00 (5.68–6.33) | 10.9 (10.5–11.3) | 18.9 (18.3–19.5) |
|  | | | | | | | | |
| **Females** | **ASD group** | | | | **Reference group** | | | |
| **Infectious diseases** | 11.5 (10.4–12.7) | 14.0 (12.8–15.2) | 18.8 (17.5–20.2) | 26.4 (24.5–28.3) | 7.14 (6.79–7.50) | 8.92 (8.53–9.32) | 13.9 (13.4–14.3) | 22.0 (21.4–22.7) |
| **Neoplasms** | 0.093 (0.027–0.264) | 0.248 (0.119–0.476) | 0.589 (0.369–0.905) | 1.52 (0.992–2.24) | 0.104 (0.067–0.158) | 0.185 (0.133–0.253) | 0.408 (0.327–0.505) | 1.37 (1.18–1.59) |
| **Blood diseases** | 1.12 (0.797–1.53) | 1.61 (1.22–2.09) | 3.36 (2.77–4.02) | 6.67 (5.58–7.88) | 0.578 (0.481–0.691) | 0.905 (0.781–1.04) | 2.07 (1.87–2.27) | 4.48 (4.15–4.83) |
| **Endocrine, nutritional, and metabolic diseases** | 3.42 (2.83–4.10) | 6.28 (5.48–7.16) | 14.9 (13.7–16.2) | 33.0 (30.8–35.3) | 1.47 (1.31–1.65) | 2.35 (2.15–2.57) | 6.02 (5.69–6.36) | 26.3 (25.5–27.1) |
| **Nervous system diseases** | 6.45 (5.64–7.33) | 10.0 (9.01–11.1) | 17.8 (16.5–19.1) | 29.4 (27.3–31.5) | 1.26 (1.11–1.42) | 2.20 (2.01–2.41) | 5.80 (5.48–6.13) | 12.5 (12.0–13.1) |
| **Diseases of the eye and adnexa** | 5.10 (4.38–5.90) | 8.37 (7.45–9.36) | 13.3 (12.2–14.5) | 19.5 (17.8–21.2) | 1.39 (1.24–1.56) | 2.48 (2.27–2.70) | 5.05 (4.75–5.36) | 9.48 (9.02–9.96) |
| **Diseases of the ear and mastoid process** | 11.3 (10.2–12.4) | 14.3 (13.1–15.5) | 17.3 (16.1–18.7) | 20.6 (19.1–22.2) | 4.65 (4.36–4.94) | 6.37 (6.04–6.72) | 8.15 (7.78–8.54) | 10.0 (9.56–10.5) |
| **Circulatory system diseases** | 0.93 (0.642–1.31) | 1.43 (1.06–1.88) | 4.78 (4.08–5.56) | 11.0 (9.65–12.5) | 0.388 (0.31–0.482) | 0.609 (0.509–0.725) | 2.35 (2.15–2.57) | 7.75 (7.29–8.22) |
| **Respiratory system diseases** | 21.9 (20.5–23.3) | 26.6 (25.0–28.1) | 33.2 (31.6–34.8) | 42.3 (40.2–44.3) | 13.3 (12.9–13.8) | 17.4 (16.9–17.9) | 25.2 (24.6–25.9) | 33.9 (33.2–34.6) |
| **Digestive system diseases** | 5.03 (4.31–5.82) | 9.40 (8.43–10.4) | 24.8 (23.3–26.3) | 39.8 (37.7–42) | 3.29 (3.05–3.55) | 5.90 (5.58–6.23) | 16.4 (15.9–17.0) | 30.4 (29.6–31.1) |
| **Skin and cutaneous system diseases** | 2.82 (2.29–3.43) | 5.08 (4.36–5.88) | 12.2 (11.1–13.4) | 22.2 (20.4–24) | 2.02 (1.83–2.22) | 3.43 (3.18–3.69) | 8.64 (8.25–9.03) | 16.8 (16.2–17.4) |
| **Musculoskeletal system diseases** | 2.63 (2.12–3.23) | 5.86 (5.08–6.70) | 24.7 (23.3–26.2) | 41.6 (39.4–43.8) | 1.40 (1.24–1.57) | 3.47 (3.22–3.73) | 20.6 (20.0–21.1) | 36.7 (36.0–37.5) |
| **Genitourinary system diseases** | 2.79 (2.26–3.40) | 5.23 (4.50–6.04) | 16.1 (14.8–17.4) | 38.8 (36.5–41.1) | 1.56 (1.39–1.74) | 2.76 (2.54–2.99) | 11.1 (10.6–11.5) | 35.8 (34.9–36.6) |
